# A31 MIRIKIZUMAB IMPROVES QUALITY OF LIFE IN MODERATELY-TO-SEVERELY ACTIVE UC: IMPROVEMENT IN IBDQ SCORES IN PARTICIPANTS OF LUCENT-1 AND LUCENT-2 RANDOMIZED, DOUBLE-BLIND, PLACEBO-CONTROLLED PHASE 3 TRIALS

**DOI:** 10.1093/jcag/gwac036.031

**Published:** 2023-03-07

**Authors:** B E Sands, B Feagan, T H Gibble, K A Traxler, N Morris, X Li, S Schreiber, V Jairath, A Armuzzi, J Jones

**Affiliations:** 1 Division of Gastroenterology, Icahn School of Medicine at Mount Sinai, New York, United States; 2 Gastroenterology, Alimentiv Inc., London, Canada; 3 Eli Lilly and Company, Indianapolis, United States; 4 University Hospital Schleswig-Holstein, Kiel, Germany; 5 Western University, London, Canada; 6 IBD Center, IRCCS Humanitas Research Hospital, Humanitas University, Milan, Italy; 7 Division of Digestive Care and Endoscopy, Department of Medicine, Department of Community Health and Epidemiology, Dalhousie University, Halifax, Canada

## Abstract

**Background:**

The inflammatory bowel disease questionnaire (IBDQ) is a measure of health-related quality of life (QoL), with higher scores indicating greater QoL. In a prior phase 2 study (NCT02589665), mirikizumab, an anti-IL23p19 antibody, demonstrated efficacy and improvement in IBDQ scores in participants with moderately to severely active ulcerative colitis (UC).

**Purpose:**

This analysis evaluated effect of mirikizumab (miri) vs placebo (PBO) on IBDQ scores in patients (pts) with moderately to severely active ulcerative colitis (UC) who had failed prior conventional or biologic therapy in a Phase 3, double-blind, 12-week (W) induction study (LUCENT-1) followed by a 40W maintenance study (LUCENT-2) for a total of 52W continuous therapy.

**Method:**

Pts (N=1162) in LUCENT-1 were randomized 3:1 to receive 300mg miri or PBO intravenously once every four weeks (Q4W). 544 pts who achieved Modified Mayo Score Clinical Response to miri by W12 of induction were rerandomized 2:1 in LUCENT-2 to subcutaneous miri 200mg or PBO Q4W in maintenance period. Randomization was stratified by previous biologic therapy failure, baseline corticosteroid use, and region. LUCENT-1 stratification included baseline (BL) disease activity, and LUCENT-2 included LUCENT-1 clinical remission status. The least squares mean change from BL in IBDQ scores at W12 of induction and W40 of maintenance was determined using analysis of covariance models. BL was W0 of therapy and stratification factors and BL scores were used as covariates. The Minimal Clinically Important Difference (MCID) was defined as an improvement of ≥16 points in total IBDQ score (IBDQ response) and IBDQ remission as a total score ≥170 points. IBDQ response and remission were calculated using non-responder imputations. Treatments were compared using the common risk difference (risk diff).

**Result(s):**

Miri treatment resulted in significantly greater improvement from BL in IBDQ total and domain scores vs PBO at both W12 of induction and W40 of maintenance (52W treatment) (Table). The proportions of pts who achieved an IBDQ response was significantly greater for miri treated pts vs PBO at W12 (risk diff =17.1[95%CI:10.7, 23.5]) and W40 (29.5 [21.0, 37.9]). Significantly greater proportions of pts receiving miri achieved IBDQ remission at W12 (18.1 [11.8, 24.4]) and W40 (28.5 [20.1, 37.0]) vs PBO (all evaluations and timepoints: p<0.001).

**Image:**

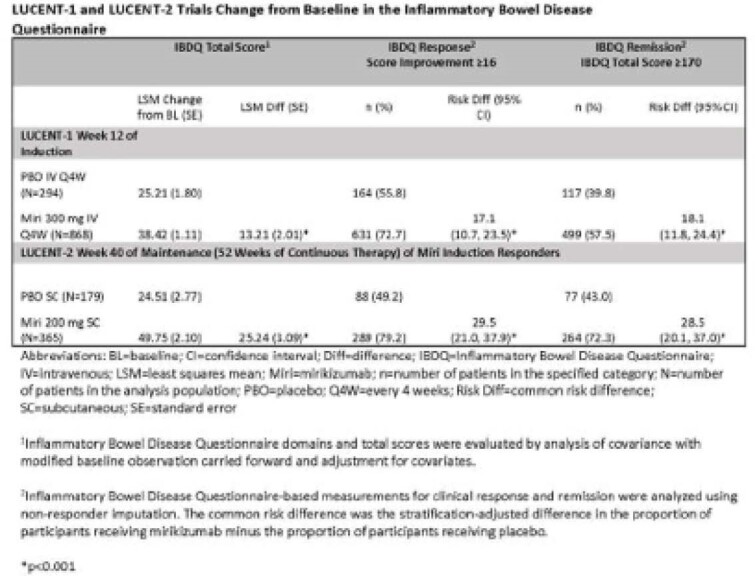

**Conclusion(s):**

Pts reported significantly greater improvements in IBDQ scores at induction and maintenance with miri compared to PBO. Over 75% of pts achieved a clinically meaningful improvement in QoL, as measured by IBDQ response, at the end of the 52 weeks of miri treatment.

**Please acknowledge all funding agencies by checking the applicable boxes below:**

Other

**Please indicate your source of funding;:**

Eli Lilly and Company

**Disclosure of Interest:**

B. Sands Consultant of: Abivax, Amgen, Arena Pharmaceuticals, Artugen Therapeutics, AstraZeneca, Bacainn Therapeutics, Boehringer Ingelheim, Boston Scientific, Bristol Myers Squibb, Calibr, Celltrion, ClostraBio, Eli Lilly and Company, Enthera, Evommune, Galapagos NV, Genentech, Gilead Sciences, GlaxoSmithKline, Gossamer Bio, InDex Pharmaceuticals, Innovation Pharmaceuticals, Inotrem, Ironwood Pharmaceuticals, Janssen, Kaleido Biosciences, Kallyope, MiroBio, Morphic Therapeutic, MRM Health, Pfizer, Progenity, Prometheus Therapeutics and Diagnostics, Protagonist Therapeutics, Q32 Bio, Surrozen, Takeda, Teva, TLL Pharmaceutical, USWM Enterprises, and Viela Bio, B. Feagan Shareholder of: Gossamer Bio, Consultant of: AbbVie, AdMIRx, AgomAb Therapeutics, Akebia Therapeutics, Alivio Therapeutics, Allakos, Amgen, Applied Molecular Transport, Arena Pharmaceuticals, Avir Pharma, Azora Therapeutics, Boehringer Ingelheim, Boston Scientific, Celgene/Bristol Myers Squibb, Connect BioPharma, Cytoki Pharma, Disc Medicine, Ecor1 Capital, Eli Lilly and Company, Equillium, Everest Clinical Research, F. Hoffmann-La Roche, Ferring Pharmaceuticals, Galapagos NV, Galen/Atlantica, Genentech/Roche, Gilead Sciences, GlaxoSmithKline, Glenmark Pharmaceuticals, Gossamer Bio, HotSpot Therapeutics, Imhotex, ImmuNext, InDex Pharmaceuticals, Intact Therapeutics, Janssen, Japan Tobacco, Kaleido Biosciences, Leadiant Biosciences, Millennium Pharmaceuticals, MiroBio, Morphic Therapeutics, Mylan, Novartis, OM Pharma, Origo Biopharma, Otsuka, Pandion Therapeutics, Pfizer, Progenity, Prometheus Therapeutics and Diagnostics, PTM Therapeutics, Q32 Bio, Rebiotix, RedHill, Biopharma, Redx Pharma, Sandoz, Sanofi, Seres Therapeutics, Surrozen, Takeda, Teva, Thelium Therapeutics, Theravance Biopharma, TiGenix, Tillotts Pharma AG, UCB Pharma, VHsquared, Viatris, Ysios Capital, and Zealand Pharma, T. Gibble Employee of: Eli Lilly and Company, K. Traxler Employee of: Eli Lilly and Company, N. Morris Employee of: Eli Lilly and Company, X. Li Employee of: Eli Lilly and Company, S. Schreiber Grant / Research support from: personal fees and/or travel support from: AbbVie, Amgen, Arena Pharmaceuticals, Biogen, Bristol Myers Squibb, Celgene, Celltrion, Eli Lilly and Company, Dr. Falk Pharma, Ferring Pharmaceuticals, Fresenius Kabi, Galapagos NV, Gilead Sciences, I-MAB Biopharma, Janssen, Merck Sharp & Dohme, Mylan, Novartis, Pfizer, Protagonist Therapeutics, Provention Bio, Roche, Sandoz/Hexal, Shire, Takeda, Theravance Biopharma, and UCB Pharma, V. Jairath Consultant of: AbbVie, Alimentiv, Arena Pharmaceuticals, Asahi Kasei Pharma, Asieris Pharmaceuticals, AstraZeneca, Bristol Myers Squibb, Celltrion, Eli Lilly and Company, Ferring Pharmaceuticals, Flagship Pioneering, Fresenius Kabi, Galapagos NV, Genentech, Gilead Sciences, GlaxoSmithKline, Janssen, Merck, Mylan, Pandion Therapeutics, Pendopharm, Pfizer, Protagonist Therapeutics, Reistone Biopharma, Roche, Sandoz, Second Genome, Shire, Takeda, Teva, Topivert, Ventyx Biosciences, and Vividion Therapeutics, A. Armuzzi Consultant of: AbbVie, Allergan, Amgen, Arena Pharmaceuticals, Biogen, Bristol Myers Squibb, Celgene, Celltrion, Eli Lilly and Company, Ferring Pharmaceuticals, Galapagos NV, Gilead Sciences, Janssen, Merck Sharp & Dohme, Mylan, Novartis, Pfizer, Protagonist Therapeutics, Roche, Samsung Bioepis, Sandoz, Takeda, and TiGenix, J. Jones: None Declared

